# Interprofessional education: A recognized necessity, a persistent challenge - Perspectives from a longitudinal study

**DOI:** 10.1371/journal.pone.0319633

**Published:** 2025-09-09

**Authors:** Thales Guardia de Barros, Emerson Roberto dos Santos, João Daniel de Souza Menezes, Matheus Querino da Silva, Marco Antonio Ribeiro Filho, Fabio Argollo Ferreira, Lucas Antonio Moura Gonzalez, Aparecida Custódio Da Silva, Izabelle Pereira Trindade, Luana Mari Takahashi, Natalia Almeida de Arnaldo Silva Rodrigues Castro, Loiane Letícia dos Santos, Camila Borge de Freitas, Natália Aparecida de Oliveira, William Donegá Martinez, Alex Bertolazzo Quitério, Ana Julia de Deus Silva, Luiz Otávio Maciel Lopes, Sônia Maria Maciel Lopes, Camila Aline Lázaro, Maria Laura Fabris, Maysa Alahmar Bianchin, Gerardo Maria de Araújo Filho, Denise Cristina Móz, Vaz Oliani, Antônio Hélio Oliani, Neuza Alves Bonifácio, Aparecida de Fátima Michelin, Vânia Maria Sabadoto Brienze, Júlio César André, Patrícia da Silva Fucuta

**Affiliations:** 1 FAMERP- Faculty of Medicine of São José do Rio Preto, Brazil; 2 Santa Casa de Misericórdia de Votuporanga, Votuporanga, São Paulo, Brazil; 3 União das Faculdades dos Grandes Lagos - UNILAGO, São José do Rio Preto, São Paulo, Brazil; 4 University Hospital Center Cova da Beira, University of Beira Interior, Covilhã, Portugal; 5 Institute of Health Sciences, Paulista University, Araçatuba, SP, Brazil; 6 Ceres School of Medicina for FACERES Medical School - São José do Rio Preto, São Paulo, Brasil; Rocky Vista University and Midwestern University, UNITED STATES OF AMERICA

## Abstract

**Background:**

Interprofessional Education (IPE) is widely recognized as essential for fostering collaborative healthcare practices and improving patient outcomes. Despite its acknowledged importance, there remains a notable scarcity of longitudinal research assessing medical students’ readiness for IPE across distinct educational stages, particularly within diverse global contexts like Brazil.

**Aim:**

This study sought to address this gap by longitudinally mapping and analyzing the evolution of medical students’ readiness for interprofessional learning throughout their academic training at a Brazilian university.

**Methods:**

Employing a quantitative longitudinal design, 53 medical students from the 2021 cohort completed the validated Readiness for Interprofessional Learning Scale (RIPLS) at three critical time points: upon university entry (2021), at the conclusion of the basic science cycle (2022), and at the end of the clinical cycle (2024). Temporal changes were assessed using repeated measures analysis of variance (ANOVA).

**Results:**

Significant global differences were observed over time in the “Teamwork and collaboration” and “Patient-centered care” dimensions. Specifically, “Patient-centered care” exhibited a non-linear pattern, characterized by an initial increase followed by a subsequent decrease. In contrast, the “Professional identity” dimension demonstrated remarkable stability across all measurement points.

**Conclusions:**

These findings reveal the complex and dynamic nature of interprofessional readiness development during medical education. They strongly advocate for the early introduction of IPE, coupled with sustained and adaptive interventions throughout the entire educational continuum, particularly to address fluctuations in patient-centered attitudes and to foster an interprofessional identity from the outset. This study offers crucial empirical insights for optimizing IPE strategies and preparing future physicians for collaborative practice.

## Introduction

Interprofessional Education (IPE) has been recognized as a promising strategy to deliver more qualified, comprehensive, and effective health care. Defined by the World Health Organization (WHO) as occasions when “two or more professions learn with, from and about each other to improve collaboration and the quality of care” [1], IPE encompasses various pedagogical approaches, including shared learning experiences, simulations, and collaborative clinical projects. The implementation of IPE often involves integrating these experiences into existing curricula, either through isolated interventions or, ideally, as a sustained, longitudinal process throughout a student’s academic journey. The growing interest in this area can be attributed to the increasing complexity of care and a better understanding of the determinants of the health-disease process, seeking alternatives to improve the quality and access to services from the perspective of comprehensive health care [2,3]. Despite its global recognition and potential benefits, effective IPE implementation faces significant challenges, often rooted in traditional educational paradigms and the complex dynamics of professional identity formation.

The performance of patient-centered interprofessional teams has proven to be more effective than care providers acting in isolation [4,5]. This collaborative model in healthcare increases the likelihood of achieving the “*quadruple aim*” of comprehensive health care: better health, better care, better value, and better work experience [6]. Effectively reorganizing healthcare services around principles of interprofessionality necessitates not only the implementation of new functional structures but also the cultivation of shared care practices and a fundamental shift in educational approaches to foster interprofessional readiness [7].

The concept of “*Readiness for Interprofessional Learning*” (RIPL) was introduced by Parsell and Bligh (1999) [8] and defined as the degree to which students are willing to engage in interprofessional learning. The Readiness for Interprofessional Learning Scale (RIPLS) has been widely used as a pre- and post-intervention test in various studies [9–11].

However, engaging healthcare students, particularly those in medicine, in interprofessional learning is not an easy task, as the traditional focus has been on developing a “*monoprofessional*” identity [12–14]. Moreover, the complexity of simultaneous teaching of different healthcare disciplines, combined with logistical problems and busy schedules, presents additional challenges for IPE implementation [15,16].

Recently, de Barros et al. (2024) [17] highlighted that readiness for interprofessional learning among medical students is a crucial factor for the success of these initiatives.

Recent research has demonstrated the positive impact of IPE on collaborative practice among healthcare professionals [18–21]. A randomized controlled trial conducted by Costa Marion et al. (2025) [22] found that interprofessional simulation practice increases readiness for collaborative practice. Lee et al. (2025) [2] emphasized the importance of educational methods such as role-play simulations, standardized patient encounters, and high-fidelity simulations in IPE.

### Theoretical framework

This study is conceptually grounded in the “Readiness for Interprofessional Learning” (RIPL) framework, originally introduced by Parsell and Bligh (1999) [8]. RIPL is defined as the degree to which students are willing and prepared to engage in learning with, from, and about other professions. This concept acknowledges that effective interprofessional education requires not only structured opportunities but also a receptiveness and predisposition among learners to participate collaboratively.

The measurement of RIPL is typically achieved through the Readiness for Interprofessional Learning Scale (RIPLS), a widely validated instrument that assesses student attitudes across key dimensions relevant to interprofessional collaboration [9–11]. Specifically, the adapted version of RIPLS utilized in this study comprises three crucial factors: Teamwork and Collaboration (F1), Professional Identity (F2), and Patient-Centered Care (F3). These factors represent distinct yet interconnected facets of readiness, reflecting both the practical skills and the underlying attitudinal shifts necessary for effective interprofessional practice.

Beyond the immediate concept of RIPL, this study also draws upon theoretical understandings of professional identity formation within medical education. Traditional medical curricula have historically emphasized the development of a strong ‘monoprofessional’ identity, often in isolation from other health professions [12–14]. This can pose a significant barrier to IPE, as it requires students to integrate new collaborative paradigms into an already forming professional self-concept. The stability or evolution of the Professional Identity (F2) dimension of RIPLS is therefore critically examined through this lens, reflecting the inherent complexity of shifting deeply ingrained professional perspectives [23,24].

Furthermore, the dynamic evolution of attitudes, particularly concerning Teamwork and Collaboration (F1) and Patient-Centered Care (F3), can be understood in the context of experiential learning theories. As students progress through basic sciences and into clinical environments, their exposure to real-world healthcare settings and collaborative practices profoundly shapes their perceptions and readiness [25,26]. The interplay between these theoretical perspectives—RIPL as a measure of predisposition, professional identity formation as a developmental process, and experiential learning as a driver of change—provides a robust framework for interpreting the longitudinal patterns observed in this study.

**Fig 1:**
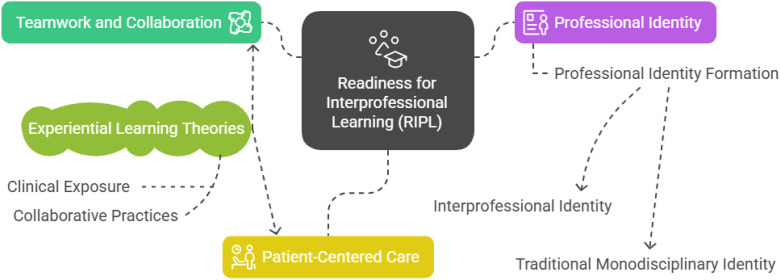
Conceptual diagram of readiness for interprofessional learning.

The integration of these theoretical perspectives establishes a comprehensive conceptual framework that elucidates the multidimensional nature of interprofessional readiness development in medical education. This framework positions RIPL as a dynamic construct influenced by the interplay between professional identity formation processes and experiential learning encounters throughout the curriculum. Within this theoretical architecture, the three RIPLS factors—Teamwork and Collaboration, Professional Identity, and Patient-Centered Care—represent interconnected yet distinct developmental dimensions, each responding differentially to educational experiences and professional socialization. The complex relationships among these constructs, including their theoretical foundations and hypothesized interactions, are systematically illustrated in the conceptual model presented in Fig 1.

This figure illustrates the overarching theoretical framework guiding the study, depicting Readiness for Interprofessional Learning (RIPL) as the central construct. It details the three primary factors of RIPL, as measured by the RIPLS (Teamwork and Collaboration, Professional Identity, and Patient-Centered Care), and their conceptual interrelations. Furthermore, the diagram highlights the influence of experiential learning theories (encompassing clinical exposure and collaborative practices) on the Teamwork and Collaboration and Patient-Centered Care factors. Concurrently, it portrays the dynamic process of professional identity formation as influencing the Professional Identity factor, delineating its potential trajectory towards either an interprofessional or a traditional monodisciplinary identity. Dashed lines indicate conceptual connections and hypothesized influences within the framework.

### Research gap, question, and hypotheses

Despite growing evidence of IPE’s benefits, a significant and critical knowledge gap persists in the literature concerning the longitudinal evolution of readiness for interprofessional learning (RIPL) among medical students. Most existing studies are cross-sectional or focus on short-term evaluations of isolated interventions, providing limited insight into how students’ readiness changes across different stages of their academic journey and the long-term effects of educational experiences. Understanding this dynamic evolution is crucial for designing sustained and effective IPE strategies, particularly given the ongoing debate about the optimal timing for IPE introduction [27–29]. Specifically, there is a scarcity of data on how the individual dimensions of RIPL—Teamwork and Collaboration, Professional Identity, and Patient-Centered Care—evolve throughout the rigorous and extended curriculum of medical education, particularly in contexts like Brazil.

Given this critical knowledge gap, the present study addresses the following research question: How does the readiness for interprofessional learning of medical students evolve throughout their academic training, specifically across different phases of their medical curriculum?

Based on existing literature and the challenges in IPE implementation, we hypothesize the following:

- H1: Readiness for Teamwork and Collaboration (F1) will show a significant, although potentially gradual, increase over time as students gain more exposure to collaborative learning environments.
- H2: Professional Identity (F2) will remain relatively stable throughout the medical curriculum, reflecting the established ‘monoprofessional’ identity formation process.
- H3: Readiness for Patient-Centered Care (F3) will exhibit a non-linear pattern, characterized by an initial increase followed by a potential decrease or stabilization as students encounter the complexities and practical realities of clinical settings [30].

### Study justification and objective

Addressing the identified gap, this study adopts a unique longitudinal approach, assessing readiness for interprofessional learning across three distinct cycles of medical education in a Brazilian institution. This comprehensive, multi-time-point perspective offers a unique opportunity to understand the intricate dynamics of RIPL evolution over time, providing insights that cross-sectional studies cannot, particularly within a Brazilian context where such longitudinal data is notably scarce.

The importance of IPE and interprofessional collaboration has long been recognized in the literature [31,32]. However, a persistent challenge remains in translating this theoretical recognition into consistently effective strategies for IPE implementation and maintenance throughout medical education, largely due to a limited understanding of the longitudinal trajectory of student readiness [33,34].

Therefore, this study is justified by its potential to provide crucial evidence regarding the dynamics of readiness for interprofessional learning over time, offering valuable insights for the development and implementation of more effective IPE strategies within medical curricula. By focusing on a longitudinal assessment, this research aims to fill a significant void in the existing literature, particularly within the Brazilian educational landscape where such comprehensive, long-term studies are rare. The longitudinal assessment across three distinct cycles (entrants, end of the basic cycle, and end of the clinical cycle) will allow for a deeper understanding of the changes in readiness for interprofessional learning throughout medical education. This may provide crucial evidence to guide the implementation and improvement of IPE initiatives, contributing to the formation of medical professionals better prepared for collaborative and interprofessional work.

Drawing on the conceptual framework of RIPL, the primary objective of this study is to longitudinally map and analyze the evolution of readiness for interprofessional learning among medical students at a Brazilian university across three distinct phases of their academic training (entry, end of basic science cycle, and end of clinical cycle), utilizing the validated Readiness for Interprofessional Learning Scale (RIPLS) instrument [35]. By detailing these temporal changes across the RIPLS dimensions—Teamwork and Collaboration, Professional Identity, and Patient-Centered Care—we aim to provide critical empirical evidence that can inform the strategic design and timing of IPE interventions, ultimately fostering the development of medical professionals who are better prepared for collaborative and patient-centered practice.

## Materials and methods

### Study design

This is a descriptive, longitudinal, and quantitative study. It is important to clarify that this study is observational in nature, aiming to map the natural evolution of medical students’ readiness for interprofessional learning (RIPL) within their existing curriculum, rather than evaluating a specific interprofessional education (IPE) intervention. This approach allows for an understanding of how attitudes and perceptions develop over time in a real-world educational setting.

### Study setting and medical program

The study was conducted at the Faculty of Medicine of São José do Rio Preto (FAMERP), a public university in Brazil. The medical program at FAMERP is a traditional six-year curriculum, structured into distinct phases: a basic science cycle (typically years 1–2), a clinical cycle (years 3–4), and an internship/clerkship phase (years 5–6). While the curriculum emphasizes patient-centered care and teamwork, it does not feature a formally structured, dedicated interprofessional education intervention. Therefore, the observed changes in RIPL reflect the influence of general academic progression, clinical exposures, and implicit learning opportunities within the standard medical training environment.

### Participants

All incoming medical students from the 2021 cohort, aged 18 years or older (with the majority being between 18 and 20 years old at the time of initial data collection), were invited to participate. The 53 participants who completed the initial assessment represented the entire cohort that consented to participate in the study. All 53 participants remained in the study and completed all three data collection points, resulting in no drop-outs from the initial participant group. Data were collected in 2021, 2022, and 2024, allowing for a comparative analysis of entrants’ data (Time 1 = entrants in 2021) with data from the end of the basic cycle in 2022 (Time 2 = 2021 entrants in 2022) and the end of the clinical cycle in 2024 (Time 3 = 2021 entrants in 2024).

### Procedures

The Informed Consent Form and research instruments were constructed using Google Forms. The access link was sent to the 2021 cohort of medical students via online channels (WhatsApp or email), following an initial presentation of the study to the cohort by the research team, facilitated by the university administration. This ensured that all students in the cohort were informed about the study and had the opportunity to participate, with additional responses collected via printed questionnaires. The estimated average time for completing the instruments was 10 minutes. The recruitment period began on March/2021 and ended on June/2021. Written or electronic informed consent was obtained from all participants before data collection.

### Instruments

The study employed two main instruments to gather data: a Sociodemographic Variables Questionnaire (QVSD) and the Readiness for Interprofessional Learning Scale (RIPLS). The selection and structuring of these questionnaires were guided by established practices in educational research and the specific objectives of this longitudinal study.

1. *Sociodemographic Variables Questionnaire (QVSD):* This instrument was designed as a custom-developed, closed-questionnaire to collect comprehensive demographic and academic background information from participants. It included items on sex, age, marital status, number of children, family income, guided physical activity, professional category, and course period. Crucially, to foster a nuanced understanding of potential influences on interprofessional readiness and to align with contemporary research trends in diversity and inclusion within healthcare education, the questionnaire also incorporated items on sexual orientation and religious affiliation [36]. The systematic collection of these variables is fundamental for thorough demographic profiling, allowing for the exploration of diverse individual trajectories and experiences that may correlate with attitudes towards interprofessional collaboration. This structured approach ensures that the sample’s representativeness can be assessed and that potential confounding demographic factors can be considered in the analysis, providing a richer context for the RIPLS scores. The complete Sociodemographic Variables Questionnaire (QVSD) will be provided as Appendix A in English.
2. *Readiness for Interprofessional Learning Scale (RIPLS):* This study utilized an adapted version of the RIPLS [37], which has been rigorously validated for the Brazilian Portuguese language [11]. The RIPLS is a widely recognized and robust psychometric instrument in interprofessional education research, comprising 27 items on a 5-point Likert scale, ranging from 1 (“Strongly Disagree”) to 5 (“Strongly Agree”). Its selection was based on its established cross-cultural validity and reliability as a gold standard for assessing students’ attitudes and perceptions regarding interprofessional learning across diverse educational and geographical contexts [8,38]. This makes it particularly suitable for longitudinal tracking of attitudinal changes, as required by our study design. While acknowledging that some literature discusses variations in its factor structure across different populations (e.g., [37], its consistent and extensive application in numerous IPE readiness studies reinforces its utility for comparative and longitudinal research, providing a consistent metric for tracking subtle shifts in readiness over time. Higher scores on each factor indicate greater readiness for interprofessional learning in that specific dimension. The adapted version consists of 3 factors:
  - Factor 1: Teamwork and collaboration (14 items)
  - Factor 2: Professional identity (8 items)
  - Factor 3: Patient-centered care (5 items).

The complete Readiness for Interprofessional Learning Scale (RIPLS) will be provided as Appendix B in English.

### Data analysis

Exploratory data analysis included descriptive statistics for numerical and categorical variables. For continuous variables, descriptive statistics, histogram and boxplot graphs, and the Shapiro-Wilk normality test were considered.

Validation of the RIPLS instrument in the sample was performed through confirmatory factor analysis, testing the model with 3 factors and 27 items. Model adjustments were verified using χ², χ²/df, RMSEA, and CFI indices. RMSEA < 0.08 and CFI ≥ 0.90 were considered acceptable [39–41]. Reliability was assessed using Cronbach’s alpha and McDonald’s omega, with a minimum acceptable value of 0.60 for McDonald’s omega.

For longitudinal analysis, repeated measures ANOVAs were conducted, testing assumptions of normality and sphericity. Normality was assessed using the Z-test for skewness and kurtosis, and sphericity by Mauchly’s test. Bootstrapping technique with 1000 samples was applied for cases of non-normal residuals.

Effect size was calculated using partial eta squared for the global ANOVA test, and Cohen’s d for paired comparisons in post-hoc tests.

### Software

All analyses were performed using R statistical software [42], with packages lavaan [43], semTools [44], nipnTK [45], bestNormalize [46], afex [47], emmeans [48], ggplot2 [49], and ggpubr [50].

### Ethical considerations

The study was approved by the Research Ethics Committee of the Faculty of Medicine of São José do Rio Preto (CEP – FAMERP), with CAAE 41220020.8.0000.5415 and opinion 4,543,158, approved on February 17, 2021. The research was conducted in accordance with the ethical principles established in the Declaration of Helsinki [51] and, in the Brazilian context, in compliance with Resolutions No. 466/2012 [52] and No. 510/2016 of the National Health Council of the Ministry of Health [53], which regulate research involving human subjects in the country. All necessary precautions were taken to ensure the confidentiality and non-identification of research participants. To ensure participant confidentiality, all data were anonymized during collection and analysis by assigning unique identification codes to each participant, and any personally identifiable information was removed from the dataset.

## Results

### Demographic characteristics

Our study cohort consisted of 53 medical students. The demographic profile indicated a predominantly young population, with the majority (65.31%) falling within the 18–20-year age bracket at the initial data collection. Regarding biological sex, males constituted a slightly higher proportion (55.10%). The majority of participants identified as heterosexual (80.61%), were single (95.92%), and reported having no children (97.96%). In terms of religious affiliation, a notable portion (44.90%) indicated no religious affiliation.

### Socioeconomic, educational, and educational history data

Analysis of socioeconomic data revealed that most participants (81.63%) reported a personal or family income between R$ 1,001.00 and R$ 3,000.00. Educational background of parents showed high levels of formal education, with most fathers (66.33%) and mothers (70.41%) having completed higher education. The vast majority of students (95.92%) were not engaged in paid employment, with only a small minority (3.06%) reporting paid work. Pertaining to their foundational education, most participants completed both primary (73.47%) and secondary (78.57%) education in private schools, with virtually all (100% for primary, 95.92% for secondary) following a regular course of study. A significant proportion (79.59%) completed secondary education within the last one to five years prior to entry into medical school.

Regarding prior higher education experiences, the overwhelming majority of participants (84.69%, n = 45) had never enrolled in another higher education course. Among those who had, 10.20% (n = 5) initiated but did not complete another course, while 7.14% (n = 4) successfully completed a prior course. Specifically, among those who left a previous course, 3.77% completed less than 50% of the course, and 1.9% completed less than one semester. Reasons for discontinuing included lack of identification with the course (1.9%) and personal preferences, such as gaining admission to Medicine (1.89%). The majority of these prior courses were undertaken at public institutions (3.787%).

Admission into the current medical program was primarily through broad competition (80.61%), followed by specific affirmative action programs: PIMESP (Programa de Inclusão com Mérito no Ensino Superior Público) for public school students (14.29%) and a quota for self-declared Black, Brown, or Indigenous students (5.10%). Most participants (65.31%) gained admission on the first call, indicating high academic preparedness. Furthermore, almost all participants (98.11%, n = 52) expressed their intention to continue in their current medical course, reflecting a strong commitment.

### Analysis of the Readiness for Interprofessional Learning Scale (RIPLS)

Table 1 presents the descriptive statistics of the dimensional scores for the three factors of the Readiness for Interprofessional Learning Scale (RIPLS): Teamwork and collaboration (F1), Professional identity (F2), and Patient-centered care (F3).

**Table 1:** Descriptive statistics of dimensional scores. (n = 53, FAMERP, 2024, Brazil).

Variables	Mean	SD	W	p-value	Min	Percentiles			Max
						P25	P50	P75	
F1	4.57	0.26	0.70	0.00	3.43	4.50	4.71	4.71	5.00
F2	2.49	0.47	0.96	0.00	1.50	2.12	2.50	2.75	4.25
F3	4.84	0.33	0.55	0.00	3.00	4.80	5.00	5.00	5.00

Note. F1: Teamwork and collaboration; F2: Professional identity; F3: Patient-centered care; W: Shapiro-test; p-value: Shapiro-test; Min: Minimum; Max: Maximum.

**Source:** Author.

For Teamwork and Collaboration (F1), the mean score was 4.57 (SD = 0.26), with scores ranging from a minimum of 3.43 to a maximum of 5.00. The Shapiro-Wilk test yielded a p-value of 0.00, indicating a non-normal distribution of scores for this factor. This finding suggests a generally high initial readiness among students in their perceptions of teamwork and collaboration, though with individual variations.

The Professional Identity factor (F2) exhibited a mean score of 2.49 (SD = 0.47), with a range from 1.50 to 4.25. Its distribution was also non-normal (Shapiro-Wilk p-value = 0.00). The 25th, 50th (median), and 75th percentiles were 2.12, 2.50, and 2.75, respectively. Notably, the mean score for F2 was considerably lower compared to F1 and F3. This lower score in professional identity suggests that while students may recognize the value of interprofessional work, their own professional self-concept may not yet fully integrate interprofessional principles, possibly reflecting the early stage of their medical training and traditional monoprofessional influences.

Patient-Centered Care (F3) showed the highest mean score at 4.84 (SD = 0.33), with scores ranging from 3.00 to 5.00. Similar to the other factors, the distribution for F3 was non-normal (Shapiro-Wilk p-value = 0.00). These high scores indicate a very strong initial disposition towards patient-centered approaches among the medical students. Overall, these descriptive findings align with Hypothesis H2, suggesting a lower initial professional identity compared to other dimensions of readiness.

### Confirmatory factor analysis and reliability

Confirmatory factor analysis of the correlation matrix was performed to validate the three-factor, 27-item model of the RIPLS within our sample. The model demonstrated acceptable fit, as indicated by the key indices: χ²(df) = 1133.926 (321); CFI = .924; RMSEA = .088 [.083,.094]. These values confirm the structural validity of the three-factor model, allowing for reliable calculation of the individual factor scores.

Reliability coefficients were assessed using Cronbach’s α and McDonald’s ω. The Teamwork and Collaboration factor (F1) showed high reliability, with Cronbach’s α = .91 and McDonald’s ω = .91. Similarly, the Patient-Centered Care factor (F3) also demonstrated strong reliability (Cronbach’s α = .85, McDonald’s ω = .85). In contrast, the Professional Identity factor (F2) presented a Cronbach’s α of.72, but a lower McDonald’s ω of.45. While Cronbach’s α for F2 is technically acceptable, the McDonald’s ω value falling below the commonly accepted threshold of 0.60 suggests moderate reliability for this specific factor in our sample, warranting cautious interpretation. This finding highlights the inherent complexity of measuring professional identity, particularly in early medical training.

Table 2 provides the descriptive statistics for the transformed factor scores, which were normalized to preserve the correlation matrix of the factors. These transformed scores show means of 0.00 for all factors, with varying standard deviations (F1 = 0.71, F2 = 0.78, F3 = 0.65) and non-normal distributions (all Shapiro-Wilk p-values = 0.00).

**Table 2:** Descriptive statistics of factor scores. (n = 53, FAMERP, 2024, Brazil).

Variables	Mean	SD	W	p-value	Min	Percentiles			Max
						P25	P50	P75	
F1	0.00	0.71	0.65	0.00	−3.60	−0.02	0.35	0.40	0.58
F2	0.00	0.78	0.62	0.00	−0.72	−0.37	−0.23	−0.06	4.96
F3	0.00	0.65	0.63	0.00	−3.78	0.09	0.15	0.23	0.94

Note. F1: Teamwork and collaboration; F2: Professional identity; F3: Patient-centered care; W: Shapiro-test; p-value: Shapiro-test; Min: Minimum; Max: Maximum.

**Source:** Author.

### Longitudinal analysis – Teamwork and Collaboration (F1)

The longitudinal analysis of the Teamwork and Collaboration factor (F1) revealed a statistically significant global difference over time (ANOVA). This finding supports Hypothesis H1, indicating that students’ readiness for teamwork and collaboration evolves throughout their medical curriculum. However, despite this overall change, post-hoc tests showed an absence of significant differences between specific time pairs (Time 1 vs. Time 2, Time 2 vs. Time 3, Time 1 vs. Time 3). This suggests a continuous and gradual, rather than abrupt, shift in attitudes towards teamwork and collaboration across the study period.

**Fig 2:**
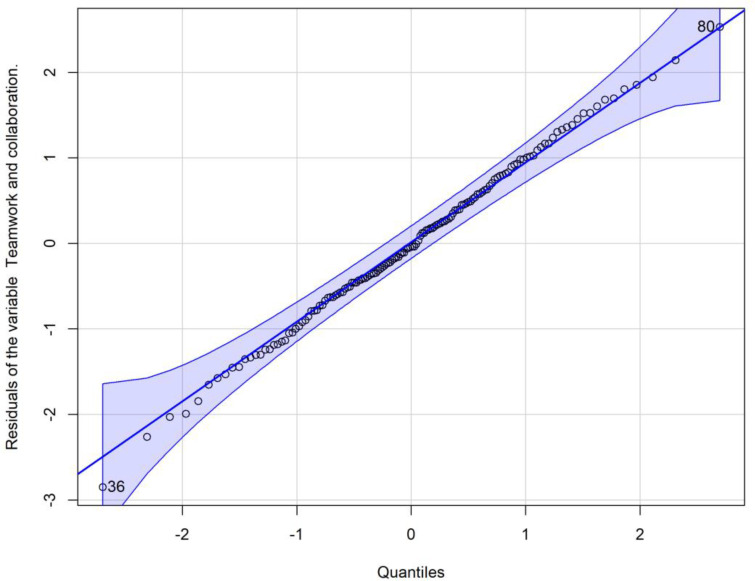
Residual plot for the Teamwork and collaboration variable. This plot displays the residuals (differences between observed and predicted values) against the fitted values for the Teamwork and collaboration factor. The red line represents the smoothed mean of the residuals, ideally close to zero, indicating a good fit of the model. (n = 53, FAMERP, 2024, Brazil). **Source:** Author.

**Fig 3:**
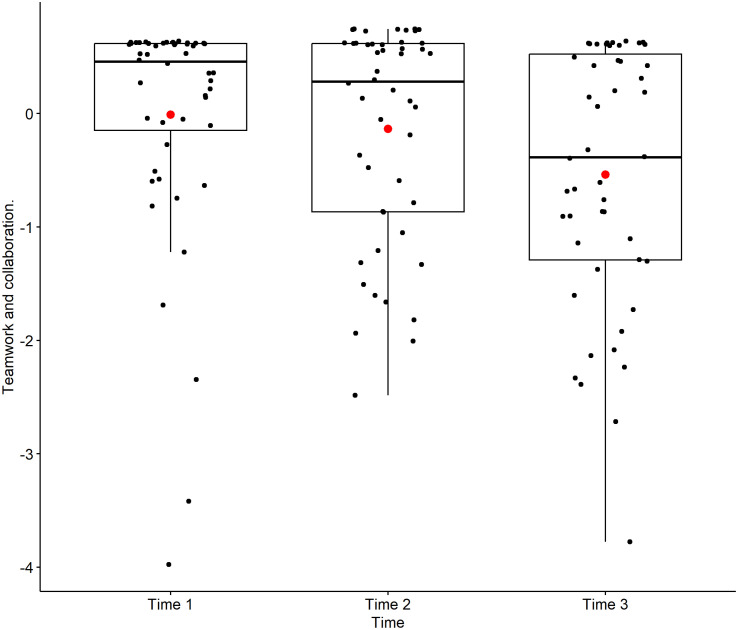
Boxplot of Teamwork and collaboration scores as a function of Time. This boxplot illustrates the distribution of scores for the Teamwork and collaboration factor at three distinct time points (Time 1: entry, Time 2: end of basic science cycle, Time 3: end of clinical cycle). The box represents the interquartile range (IQR), the line inside the box is the median, and the whiskers extend to 1.5 times the IQR. The red dot within each box indicates the mean score for that time point. (n = 53, FAMERP, 2024, Brazil). **Source:** Author.

Fig 2 illustrates the residual plot for the Teamwork and Collaboration variable. The red line, representing the smoothed mean of the residuals, is ideally close to zero, which visually confirms a good fit of the model. Fig 3, a boxplot of Teamwork and Collaboration scores across the three time points (Time 1: entry, Time 2: end of basic science cycle, Time 3: end of clinical cycle), visually supports the finding of a continuous evolution. While the mean scores (red dots within each box) show a slight upward trend, the overlapping interquartile ranges (boxes) and whiskers across time points underscore the lack of significant pairwise differences. The distribution, as depicted by the boxes, indicates that scores generally remained high over time, suggesting an enduring positive inclination towards teamwork among students.

### Longitudinal analysis – Professional Identity (F2)

The longitudinal analysis of the Professional Identity factor (F2) revealed no statistically significant difference over time (ANOVA). This finding provides strong support for Hypothesis H2, which posited that Professional Identity would remain relatively stable throughout the medical curriculum. The residuals for this analysis were normally distributed. This stability suggests that the core aspects of professional identity, particularly as measured by RIPLS, are established relatively early in medical training and do not undergo substantial statistical shifts across the observed phases of the curriculum.

### Longitudinal analysis – Patient-Centered Care (F3)

The longitudinal analysis of the Patient-Centered Care factor (F3) demonstrated a statistically significant global difference over time (ANOVA). Post-hoc analysis revealed a significant increase in scores between Time 1 (entry) and Time 2 (end of basic science cycle), followed by a significant decrease in scores between Time 2 and Time 3 (end of clinical cycle). This non-linear pattern, characterized by an initial rise and subsequent decline, strongly supports Hypothesis H3, indicating that students’ attitudes towards patient-centered care undergo a dynamic and fluctuating evolution during their medical training.

**Fig 4:**
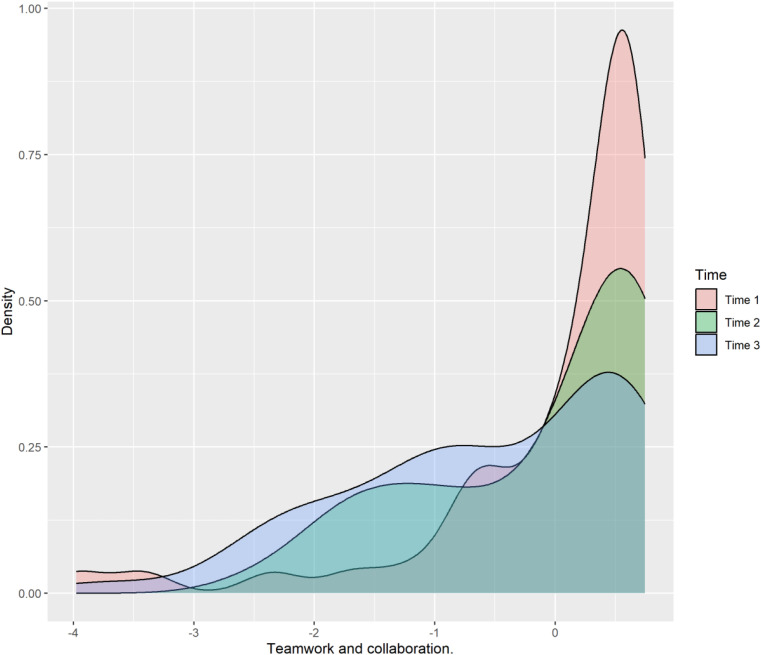
Distribution of Patient-centered care scores as a function of Time. This plot (likely a density plot or violin plot) illustrates the probability density of scores for the Patient-centered care factor across the three time points (Time 1: entry, Time 2: end of basic science cycle, Time 3: end of clinical cycle), providing a visual representation of the score distribution at each stage. (n = 53, FAMERP, 2024, Brazil). **Source:** Author.

**Fig 5:**
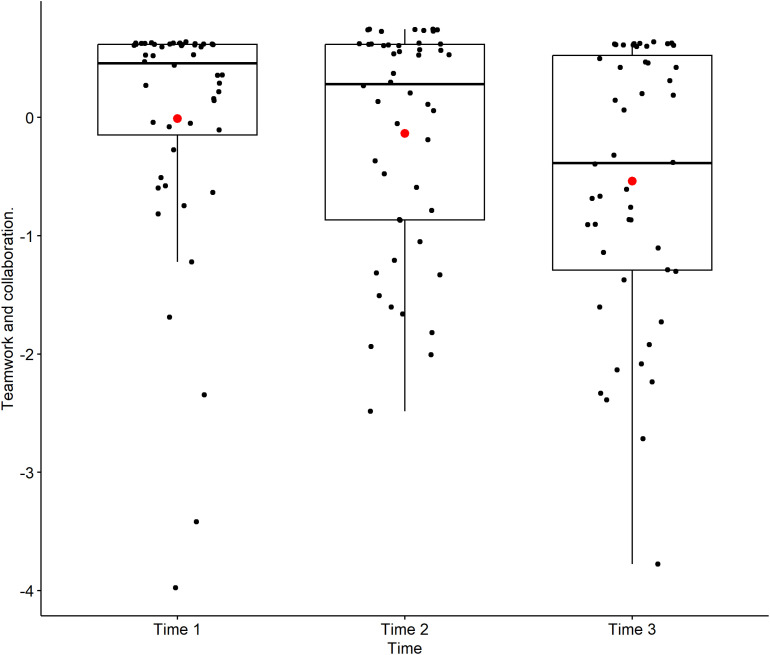
Boxplot of Patient-centered care scores as a function of Time. This boxplot illustrates the distribution of scores for the Patient-centered care factor at three distinct time points (Time 1: entry, Time 2: end of basic science cycle, Time 3: end of clinical cycle). The box represents the interquartile range (IQR), the line inside the box is the median, and the whiskers extend to 1.5 times the IQR. The red dot within each box indicates the mean score for that time point. (n = 53, FAMERP, 2024, Brazil). **Source:** Author.

The residuals for this factor exhibited a non-normal distribution, necessitating the use of a bootstrapping technique for robust analysis, as detailed in the Methods section. Fig 4, which illustrates the probability density of scores for Patient-Centered Care across the three time points, visually emphasizes this non-linear pattern. The distribution widens and shifts between time points, particularly between Time 2 and Time 3, reflecting the observed fluctuations. Fig 5, a boxplot of Patient-Centered Care scores, further visually reinforces this “inverted U” pattern. The mean score (red dot) is highest at Time 2, followed by a decrease at Time 3, while still remaining above the Time 1 mean. The boxplots also show changes in spread and median position, confirming the dynamic evolution and the challenges students may face in maintaining consistently high patient-centeredness as they progress into clinical realities.

## Discussion

This longitudinal study provides a comprehensive understanding of the evolution of readiness for interprofessional learning (RIPL) among medical students at a Brazilian university, directly addressing our aim to map these dynamic changes across different phases of their academic training. Our findings reveal a complex interplay of evolving attitudes towards teamwork and patient-centered care, alongside a notable stability in professional identity formation. This nuanced trajectory offers critical insights for the strategic design and implementation of interprofessional education (IPE) initiatives, particularly within contexts like Brazil where such longitudinal evidence remains scarce.

### Demographic and academic profile of students: Implications for medical education and interprofessional education

The demographic profile of our medical student sample, predominantly young, single, and without children, reflects a cohort primarily dedicated to intensive academic pursuits. This early entry into medical school and focus on studies aligns with a life stage conducive to rigorous professional training.

While the observed slight male majority contrasts with broader feminization trends in higher education, this demographic composition provides crucial context for understanding students’ receptiveness to new educational experiences, including IPE. Exposure to diverse perspectives, such as those related to religiosity and sexual orientation, can foster a better understanding and acceptance of teamwork and collaboration in healthcare [25]. The strong family investment in education, evidenced by high parental educational levels despite moderate family incomes, underscores a significant cultural capital that likely drives students’ academic aspirations and commitment to medical training [5,54]. This dedication, coupled with a history of private schooling and rapid entry into university, suggests a solid academic foundation. However, this traditional educational background may also present challenges for integrating more humanistic and interprofessional approaches, potentially reinforcing a monoprofessional identity [55]. The high competitiveness of the admission process and students’ strong intention to continue in the course indicate a highly motivated and committed student body, which can be a positive factor for implementing innovative educational approaches like IPE, provided they are presented as integral to modern medical practice [25].

### Longitudinal evolution of RIPLS dimensions: Dynamics and challenges in interprofessional medical education

Our analysis of the Readiness for Interprofessional Learning Scale (RIPLS) revealed distinct patterns among participants that evolve throughout their medical curriculum. Initial high scores in “Teamwork and collaboration” (F1) and “Patient-centered care” (F3) indicated a strong early disposition towards collaborative work and patient-centered approaches, consistent with findings by Zaher et al. (2022) [25]. This suggests an early recognition of IPE’s importance among medical students. Conversely, the “Professional identity” dimension (F2) presented considerably lower initial scores, reflecting the inherent complexity of professional identity formation in early medical training. This discrepancy aligns with observations that medical students may face challenges integrating teamwork and patient-centered concepts with their developing professional self-concept, possibly due to traditionally hierarchical training models [54,56]. The observed non-normal distribution across all factors indicated a notable heterogeneity in student responses, suggesting diverse individual trajectories and experiences influencing their readiness for interprofessional learning. These initial findings underscore the importance of integrating IPE strategies from the outset of medical education to cultivate a collaborative culture and foster a professional identity that embraces interprofessional principles [57,58].

### Professional Identity (F2): Remarkable stability and its implications

In contrast to the evolving nature of F1, the “Professional identity” dimension exhibited remarkable stability over time, with no statistically significant changes detected across the three measurement points. This robustness in identity construction from early stages provides strong support for Hypothesis H2, which posited that professional identity would remain relatively stable throughout the medical curriculum. This stability suggests that while students may express openness to collaboration, their core professional identity, heavily influenced by traditional medical curricula, is established relatively early and resists substantial statistical shifts across the observed phases [12,13]. This finding prompts a critical reflection: does this stability indicate an early and firm entrenchment of a monoprofessional identity, potentially hindering the full integration of interprofessional principles? Or does it highlight a need for more targeted pedagogical strategies that consciously aim to reshape professional identity from the outset to be more interprofessionally inclusive [59,60]? The absence of significant change underscores the profound challenge of altering deeply ingrained professional self-concepts later in training, suggesting that early, intentional interventions are crucial to foster a truly interprofessional identity.

### Patient-Centered Care (F3): A non-linear, “Inverted U” pattern

The “Patient-centered care” dimension displayed a dynamic and non-linear, “inverted U” pattern. We observed a significant increase in scores between Time 1 (entry) and Time 2 (end of basic science cycle), followed by a significant decrease between Time 2 and Time 3 (end of clinical cycle). This fluctuation strongly supports Hypothesis H3, indicating that students’ attitudes towards patient-centered care undergo a complex evolution during their medical training. The initial rise likely reflects an early idealism and growing exposure to patient-centered concepts in foundational coursework. However, the subsequent decline during the clinical cycle suggests that this idealism may be challenged by the practical complexities and realities of real-world clinical settings [61,62]. This pattern highlights that maintaining consistently positive attitudes towards patient-centered care requires continuous reinforcement and specific educational interventions at different stages of the medical course, particularly as students transition into the more demanding and often fragmented clinical environments [63,64]. The non-normal distribution of residuals for this factor further underscores the varied individual experiences and challenges students face in developing and sustaining this crucial competency.

### Implications for medical education

The complex and dynamic patterns observed in “Teamwork and collaboration,” “Professional identity,” and “Patient-centered care” have significant implications for medical education, offering concrete directions for enhancing interprofessional competencies. Firstly, the evolving nature of Teamwork and Collaboration readiness, coupled with the “inverted U” pattern in Patient-Centered Care, clearly indicates a necessity for sustained and continuous educational interventions throughout the medical curriculum. These interventions must be adaptive, particularly during critical transition periods between basic and clinical cycles, to maintain and reinforce positive attitudes towards interprofessional collaboration and patient-centered care [28].

Secondly, the remarkable stability observed in professional identity formation highlights a critical area for targeted pedagogical strategies. Instead of viewing professional identity as an immutable trait, medical curricula must specifically address the formation of an interprofessional identity from the outset [29]. This can be achieved through early and consistent exposure to diverse healthcare roles, team-based learning, and reflective practices that challenge monoprofessional perspectives, thereby shaping a professional self-concept that naturally embraces collaborative principles [31,32].

Finally, these findings emphasize the need for a holistic curriculum review to embed more structured opportunities for interaction and learning with students from other health professions. Investing in faculty development is equally crucial to equip educators with the skills and mindset to effectively facilitate and promote these interprofessional learning experiences, moving beyond isolated interventions to fully integrated, curriculum-wide approaches [27]. This study’s contribution lies in providing longitudinal empirical evidence, particularly from a Brazilian context, to guide these strategic shifts, fostering the development of medical professionals better prepared for collaborative and patient-centered practice in increasingly complex healthcare systems.

## Limitations

This study has several limitations that warrant consideration. Firstly, the sample size of 53 participants, while allowing for longitudinal analysis, is relatively small and drawn from a single institution in Brazil. This limits the generalizability of our findings to other medical schools or diverse cultural contexts. Secondly, the study relied on a self-report instrument (RIPLS), which may be subject to social desirability bias. While RIPLS is a validated tool, it captures self-perceived readiness rather than directly observed collaborative behaviors or competencies. Thirdly, the quantitative design, while effective for mapping trends, does not fully capture the underlying reasons or specific experiences that contribute to the observed changes (or lack thereof) in students’ readiness. We did not directly assess the specific IPE interventions or pedagogical methods implemented during the study period, nor did we collect explicit student perceptions of these implementations. Finally, the absence of a control group means that observed changes cannot be definitively attributed solely to the curriculum or specific educational experiences, as other external factors might also play a role.

### Future perspectives

Building on these findings, future research should pursue several avenues to further advance the understanding and implementation of IPE:

1. **Multi-institutional Studies:** Conduct similar longitudinal studies in other institutions and diverse cultural contexts to validate and expand these findings, enhancing generalizability.
2. **Influencing Factors:** Investigate specific factors (e.g., curriculum design, specific IPE activities, clinical environment, individual student characteristics) that influence fluctuations in readiness for interprofessional learning over time, particularly for patient-centered care.
3. **Targeted Interventions:** Develop and evaluate targeted educational interventions designed to improve readiness for interprofessional learning at critical points in medical training, particularly addressing the “inverted U” pattern in patient-centered care.
4. **Post-Graduation Outcomes:** Explore the relationship between readiness for interprofessional learning during medical school and practical outcomes in professional collaboration after graduation.
5. **Implementation Models:** Investigate the impact of different models of implementing interprofessional education (e.g., immersion vs. exposure) on student readiness.
6. **Comparative Studies:** Conduct comparative studies between different health professions to identify similarities and differences in the evolution of readiness for interprofessional learning.
7. Refined Assessment: Develop more refined assessment instruments that can capture the nuances of readiness for interprofessional learning over time, potentially incorporating qualitative measures of student perceptions and experiences.

These conclusions, implications, and future perspectives reflect the complexity and importance of the topic, highlighting the need for a continuous and adaptive approach in promoting interprofessional education in medical training.

## Conclusions

This longitudinal study successfully mapped the complex, dynamic evolution of readiness for interprofessional learning (RIPL) among medical students in a Brazilian university, thereby addressing a critical gap in the existing literature, particularly within this specific context. Our findings reveal a nuanced trajectory across the three key dimensions of RIPL: ‘Teamwork and collaboration’ (F1) demonstrated a significant, gradual evolution over time, indicating a continuous influence of academic and clinical experiences on students’ propensity for collaborative practice. In stark contrast, ‘Professional identity’ (F2) exhibited remarkable stability throughout the medical curriculum, suggesting that core professional self-concepts are established relatively early and resist substantial attitudinal shifts. Furthermore, ‘Patient-centered care’ (F3) displayed a dynamic ‘inverted U’ pattern, characterized by an initial increase followed by a significant decrease during the clinical cycle, underscoring the challenges students face in maintaining consistently high patient-centeredness amidst the complexities of real-world practice.

These patterns underscore several critical implications for medical education. The continuous evolution of teamwork readiness and the fluctuating nature of patient-centered attitudes highlight the imperative for sustained, adaptive, and strategically timed interprofessional education (IPE) interventions across the entire curriculum. Notably, the observed stability in professional identity formation signals a pressing need for pedagogical strategies that actively cultivate an interprofessional identity from the earliest stages of medical training, rather than attempting to reshape deeply ingrained monoprofessional perspectives later. Such strategies should include early and consistent exposure to diverse healthcare roles and team-based learning, coupled with continuous reinforcement of patient-centered principles, especially as students transition into demanding clinical environments.

While this study provides valuable longitudinal insights, it is important to acknowledge its limitations, including a relatively small, single-institution sample and reliance on self-report data, which may restrict generalizability. Despite these considerations, our research contributes crucial empirical evidence to inform the strategic design of IPE initiatives, curriculum reform, and faculty development efforts. Ultimately, this study supports the formation of medical professionals better equipped for collaborative, patient-centered care within modern healthcare systems. Future research should build upon these findings through multi-institutional studies, deeper exploration of influencing factors, and the development and evaluation of targeted IPE interventions to address the specific challenges identified herein.
